# Treatment Needs for Women With Alcohol Problems

**Published:** 1994

**Authors:** Linda J. Beckman

**Affiliations:** Linda J. Beckman, Ph.D., is a professor at the California School of Professional Psychology, Los Angeles, California

## Abstract

To provide effective treatment for women with alcohol problems, specific risk factors for and consequences of women’s drinking must be recognized and addressed in treatment programs. Women-sensitive treatment components can be implemented in mixed-gender as well as in women-only settings.

Do women require alcoholism treatment that differs from that for men? And if they do, what components should be included in women-oriented treatment? Until now, these questions have generated much rhetoric but few empirical data. Researchers and service providers have designed treatment components for female patients, incorporating results from clinical and survey studies of women with alcohol problems.^1^ Yet the effects of these approaches on treatment outcome rarely are evaluated directly. For example, because investigators have found that alcoholic women tend to have lower self-esteem than do alcoholic men (Beckman 1994), some programs have developed treatment components designed to bolster self-esteem. Yet the effects of these components, by themselves or in combination with other treatment services, on women’s treatment outcome or even on increasing self-esteem have not been studied adequately.

This article reviews the characteristics of women-sensitive alcoholism treatment and discusses specific aspects and risk factors for women’s alcohol abuse that should be addressed in a women-sensitive treatment program. It also examines differences in treatment outcomes for women and men and considers data on the effectiveness of women-only treatment programs.

## Designing Treatment Programs for Women

Some of the causes and consequences of alcohol abuse may differ between women and men, thereby necessitating gender-specific treatment approaches. Yet women with drinking problems are not a homogeneous group with homogeneous needs. They fall into subgroups with specific characteristics, such as age, ethnicity, sexual orientation, prior experiences of violence and sexual abuse, and type and severity of symptoms. These subgroup differences also can have implications for treatment content.

To receive optimal treatment, patients should be matched to treatment approaches that recognize their specific problems and lifestyles (Lindstrom 1992). In certain cases, this patient-treatment matching may require separate treatment programs for subgroups of alcohol-abusing women (e.g., pregnant women and minority women). More often, an individualized treatment plan can be developed that, within the general program, combines specific treatment components and intensities for each patient. In the age of managed care, this approach may provide the most effective combination of services to women at the lowest cost.

### What Is Women-Oriented Alcoholism Treatment?

Several characteristics define a women-oriented treatment program (Reed 1987):

- It is delivered in a setting that is compatible with women’s interactional styles and personal orientations; for example, it takes into consideration women’s need for and responsiveness to social relationships.
- It takes into account gender roles, female socialization (i.e., how women are brought up to occupy traditionally female roles in family and society), and women’s status in society (e.g., many areas of society and family have a patriarchal power structure).
- It does not exploit women; for example, it does not allow sexual harassment of female patients nor does it support passive, dependent roles for women.
- It addresses women-specific treatment issues.

Such a treatment philosophy can be incorporated into a mixed-gender program but may require women-only groups, female therapists, or both as part of the treatment.

A women-oriented treatment approach requires the availability of a broad range of coordinated treatment components (see box). These include family services, chemical dependency education, development of parenting skills, training to develop self-esteem and coping mechanisms, vocational/educational counseling and training, and legal assistance (Reed 1987). Women’s use of alcoholism services and treatment outcome also are likely to be improved by the availability of additional services, which may remove barriers to women’s treatment entry (see sidebar). These include child care, counseling for children, women’s support groups, health care during treatment, and support services after completion of the formal treatment program.

**Components of Women-Oriented Alcoholism Treatment**

Broad and comprehensive services

Treatment for other problems:

Incest, sexual assault, and violence
Sexual dysfunction
Multiple substance abuse
Other mental health problems

Health services

Family services

Services for children

Development of parenting skills

Development of social roles, positive relationships, and social support

Development of self-esteem and adaptive coping mechanisms

Employment/vocational counseling

Legal assistance

Women’s support groups as aftercare

**Barriers to Alcoholism Treatment for Women**

Women still are underrepresented in alcoholism treatment. Recent national surveys estimated that 25 to 30 percent of people with alcohol problems are women (Wilsnack in press). Yet according to Government surveys in the late 1980’s, women made up only about 20 percent of patients in alcoholism treatment programs (Blume in press).

Women face many gender-specific barriers that may limit their access to alcoholism treatment (see box). These barriers can be both internal (i.e., reflecting the woman’s own perception of her drinking problem) and external (i.e., exerted by the woman’s environment). The latter can be differentiated further into interpersonal and structural barriers.

**Barriers to Alcoholism Treatment for Women**

***Internal Barriers***

Denial of drinking problem

Fear of stigmatization

Concern about leaving or losing children

Guilt and shame

***External Barriers***

Interpersonal Barriers

Opposition by family and friends
Social costs of treatment

Structural Barriers

Different referral patterns
Inadequate training of health professionals
Lack of women-sensitive treatment services
Lack of economic resources
Inadequate insurance coverage
Lack of child care facilities

***Internal Barriers***

Alcohol-abusing women often have a different perception of their problem than do alcohol-abusing men. Women frequently do not believe that drinking is their main problem but instead perceive alcohol use as a coping response to a specific crisis or to problematic social situations (Reed 1987; Thom 1986). Gomberg (1993) reported that the most frequent reasons given by women for seeking treatment were depression; medical problems related to alcohol use; problems with partner, spouse, or children; and, especially among middle-age women, “empty nest” situations related to children leaving home.

Because of this different perception, women are less likely than men to seek help initially in alcoholism or other chemical dependency services. Instead, women prefer consulting physicians or mental health clinics staff, settings in which their primary problem is less likely to be diagnosed as alcohol abuse (Reed 1987; Thom 1986). It is critical that the women’s alcohol problems be identified correctly in these settings and that appropriate interventions be initiated.

There still is a greater social stigma associated with alcohol abuse for women than for men (Reed 1987). The resulting reluctance to be labeled as “alcoholic” may cause women to deny or minimize their drinking problems and to delay seeking treatment (Wilsnack in press).

Women may avoid treatment because they do not want to leave their children or do not have the resources to arrange for independent child care. They also may fear that their children would be taken away from them because of their addiction (Wilsnack in press).

Finally, women often delay entering alcoholism treatment because of feelings of guilt and shame. They consider admitting to an alcohol problem as an indication of their failure to fulfill adequately the roles of mother, wife, and/or sexual partner (Beckman and Amaro 1986).

***External Barriers***

***Interpersonal Barriers***. Women with drinking problems generally seem to experience more barriers in their social environments than do men. According to one study, family and friends are more likely to oppose the treatment entry of white women than of white men (Beckman and Amaro 1986). Social stigma, economic concerns, and perceived conflict with family roles may be reasons underlying this lack of support.

The opposition of family members to treatment and, consequently, the disruption of family relationships are part of the “social costs” associated by many women with alcoholism treatment. Other factors, such as avoidance by friends or coworkers and feelings of loneliness, also may discourage women from seeking help.

***Structural Barriers***. Women’s entry into alcoholism treatment also can be hindered by their referral patterns, which are different from those of men (Duckert 1987). Women are less likely than men to be referred through conventional routes, such as physicians, the legal system, and employers. More often, they are referred by family or friends and learn about specific programs through advertisements or word of mouth (Beckman and Kocel 1982).

Physicians would seem an excellent source of referral, yet at least one large study found them to be less effective in identifying alcohol abuse in women than in men (Moore et al. 1989). This highlights the need to train health care providers to diagnose, treat, and refer competently women with alcohol problems.

Women may be kept from seeking treatment by the lack of women-sensitive treatment in existing programs. Studies have shown that the proportion of female clients is greater in programs that provide services for children and followup care and that have more women on their professional staff than in programs without these characteristics (Beckman and Kocel 1982).

Some women also experience economic barriers to treatment. These barriers include low income (which may not allow them to arrange for independent child care) and inadequate insurance coverage for alcoholism treatment services.

***Subgroup Differences***. The predominant barriers can vary for different subgroups of women. For example, for white women, the personal and social costs associated with treatment entry are an important factor (Beckman and Amaro 1986). African-American women, on the other hand, are more influenced by structural (particularly economic) than by interpersonal factors (Beckman 1994).

***How Can Treatment Access for Women Be Improved?***

To remove the barriers that restrict women’s access to alcoholism treatment, several measures can be taken:

- Educate the general public. Such education is essential for removing individual and interpersonal barriers based on the stigma against alcohol-abusing women.
- Increase the knowledge about women’s drinking problems to improve detection and referral by physicians, the legal system, and employee assistance programs. Informal referral networks, such as friends and family members, also should be used more efficiently.
- Implement routine alcoholism screening programs in pregnancy and family planning clinics and obstetrician/ gynecologists’ offices to aid in the identification of women with alcohol problems.
- Improve the availability of services for children. These services may include supervision while the woman is participating in treatment activities, diagnosis and treatment for children with behavioral problems, and education about the mother’s alcoholism.
- Increase the availability of other women-sensitive services that address gender-specific problems and treatment issues and that consider women’s social contexts. It also is important for outreach efforts to highlight such characteristics when they are present in treatment services.
- Improve the outreach to women at risk for drinking problems. Known risk factors include a family history of alcoholism; a heavy-drinking partner; drinking patterns that are discrepant from those of the partner; cohabitation; lack of social roles, loss of social roles, or involuntary social roles; depression and anxiety; childhood sexual or physical abuse; physical violence or sexual assault in adulthood; and sexual problems (for more information, see the article by Wilsnack et al., pp. 173–181).
  —*Linda J. Beckman*

ReferencesBeckmanLJEntry of women of diverse backgrounds into alcoholism treatmentLewisJAddictions: Concepts and Strategies for TreatmentGaithersburg, MDAspen Publishers1994365374BeckmanLJAmaroHPersonal and social difficulties faced by women and men entering alcoholism treatmentJournal of Studies on Alcohol4721351451986371317510.15288/jsa.1986.47.135BeckmanLJKocelKMThe treatment-delivery system and alcohol abuse in women: Social policy implicationsJournal of Social Issues3821391511982BlumeSBWomen and alcohol: Issues in social policyWilsnackRWWilsnackSCGender and AlcoholNew Brunswick, NJRutgers University Center of Alcohol Studiesin pressDuckertFRecruitment into treatment and effects of treatment for female problem drinkersAddictive Behaviors1221371501987363080010.1016/0306-4603(87)90020-7GombergESLWomen and alcohol: Use and abuseJournal of Nervous and Mental Disease18142112191993847387210.1097/00005053-199304000-00001MooreRDBoneLRGellerGMamonJAStokesEJLevineDMPrevalence, detection, and treatment of alcoholism in hospitalized patientsJournal of the American Medical Association261340340719892909780ReedBGDeveloping women-sensitive drug dependence treatment services: Why so difficult?Journal of Psychoactive Drugs1921511641987361238310.1080/02791072.1987.10472399ThomBSex differences in help-seeking for alcohol problems: 1. The barriers to help-seekingBritish Journal of Addiction8167777881986346777710.1111/j.1360-0443.1986.tb00405.xWilsnackSCAlcohol use and alcohol problems in womenStantonALGallantSJPsychology of Women’s Health: Progress and Challenges in Research and ApplicationWashington, DCAmerican Psychological Associationin press

### How Can Treatment Be Made Sensitive to Women?

What practical steps can be taken to implement a women-sensitive program? Gomberg (1993) noted that “the most critical aspect of treatment for alcoholic women lies in therapists’ attitudes toward women in general and toward substance-abusing and alcoholic women in particular” (p. 218). Therefore, therapists and staff members must be aware of and sensitive to women’s problems, treatment requirements, and status in society.

Other characteristics and practices of a treatment program also can be made more sensitive and satisfying to women (Reed 1987). For example, the physical environment (e.g., furniture and decorations), interaction patterns of staff and clients, and intake procedures may have to be adapted to women’s preferences. Integration of diverse sources of social support into treatment programs also can help to make the program more sensitive to women.

## Women-Specific Treatment Components

Women’s needs in alcoholism treatment fall into three categories. The first category is unique to women, such as issues related to female physiology and reproductive functions. Certain aspects of psychological growth and development also may be gender specific. The second category covers treatment needs more relevant for women than for men. For example, female problem drinkers are more frequently the victims of sexual abuse or physical violence than are their male counterparts. The third category includes problems that are relevant to both women and men, such as psychiatric comorbidity and multiple substance abuse.

### Reproductive Health and Sexual Problems

Excessive drinking^2^ can adversely affect the menstrual cycle, fetal development, and childbirth; can lead to changes in sexual desire, such as sexual inhibition and lack of sexual interest; and has been linked to early menopause (Gomberg 1993). Sexual problems are very common among women in alcoholism treatment. Prevalence estimates range from about 20 to 100 percent, depending on sample characteristics and definitions and assessment of sexual problems (Beckman and Ackerman in press).

Some female problem drinkers use alcohol to cope with preexisting sexual problems; for example, to heighten low sexual desire or to decrease sexual inhibition (Beckman and Ackerman in press; see the article by Wilsnack et al., pp. 173–181). Alcohol consumption, however, may lead to even greater sexual problems (Beckman and Ackerman in press). Women problem drinkers therefore need education and counseling on how to break this cycle.

Because sexual problems are common among female problem drinkers, it is important that treatment programs identify and assess the type and severity of problems experienced and provide specialized treatment if necessary. To provide these services, treatment programs may use staff members with advanced training in treatment of sexual problems or may collaborate with sex therapists or other specialized health care professionals. Sexually active women also may need contraceptive and reproductive counseling as well as education on how to communicate with sexual partners about use of condoms and other contraceptives and how to avoid risky sexual behavior (e.g., sexual intercourse without a condom or with multiple partners).

### Sexual Assault and Physical Abuse

The rates of sexual abuse and incest as children or adults are significantly higher among women with drinking problems than in the general population. Estimates of the prevalence of incest, for example, range from 12 to 85 percent for alcoholic women, depending on the populations and definitions of incest used in different studies (Beckman and Ackerman in press). Incest rates among nonalcoholic women in each studied population generally were lower than among the alcoholic women. For abused women, drinking initially may have been a way of coping with the trauma associated with incest or sexual assault (Beckman and Ackerman in press).

Women problem drinkers also are more likely than other women to have experienced physical violence, both as children and as adults (Miller et al. 1989, 1993). Some women still are in violent relationships when they enter alcoholism treatment. For these women, fear of their partner’s reprisals may adversely affect treatment outcome and therefore has to be considered during treatment.

The sexual and physical violence experienced by women sometimes can lead to severe mental health consequences, including posttraumatic stress disorder (PTSD), a condition associated with extremely stressful situations, such as war or disasters. Symptoms of PTSD include flashbacks, intrusive thoughts, sleeping and concentration problems, and persistent avoidance of stimuli associated with the traumatic events.

Specific treatment for the consequences of sexual abuse and physical violence, including PTSD, is necessary for many women with alcohol problems. In addition to counseling, some women may need shelters or inpatient programs that offer safe havens from abusive partners and treatment that provides them with the confidence, understanding, and skills necessary to leave abusive relationships.

### Social Support Systems

Development of new social roles and relationships that provide a social support network, as well as strengthen the current support network, is an important aspect of alcoholism treatment for women. The literature on women’s psychological development emphasizes the central role in women’s lives of relationships with others and postulates a greater importance of these social connections for women than for men (e.g., Miller 1984).

One aspect of social connections is the giving and receiving of support, and alcoholic women appear to receive less support from their families and friends than do nonalcoholic women, both as children and as adults (Gomberg 1993). The importance of social networks also is underscored by survey data showing that women who lack some social roles (e.g., women who are unmarried or who are without full-time work) or have lost them (e.g., through divorce or the “empty nest” syndrome) report more problem-drinking indicators than do those with multiple roles (see the article by Wilsnack et al., pp. 173–181).

Although the number of supportive relationships has been shown to be a significant predictor of treatment outcome for women alcoholics (Macdonald 1987), the quality of these relationships, for example with partners and children, can be an even more important factor (Beckman 1981). Partners can encourage women’s drinking and undermine treatment or can serve as a powerful motivator for abstinence. Similarly, women’s concern for their children can hinder their treatment (e.g., if adequate child care is not available) or can motivate them to overcome their drinking problems. Therefore, the development of satisfying and supportive intimate friendships, relationships, and family ties is an important therapeutic goal for women with drinking problems.

It is noteworthy that different ethnic groups may receive different levels of support from their social networks. For instance, African-American women who enter treatment have stronger support from their families than do white women (Beckman 1994).

### Psychiatric Comorbidity

Almost two-thirds of female problem drinkers and alcoholics have multiple mental health problems (Helzer and Pryzbeck 1988). It is not clear whether the prevalence of comorbid psychiatric disorders differs in male and female alcoholics. Until recently, the literature consistently reported more psychiatric disorders among women than among men, but at least one recent study contradicts this finding (see Gomberg 1993).

Women and men do differ in the prevalence of specific psychiatric disorders. In a large multisite community study, Helzer and Pryzbeck (1988) found that alcohol-abusing women were more likely than alcohol-abusing men to have secondary diagnoses of mania, somatization (i.e., the conversion of anxiety into physical symptoms), major depression, phobic disorder, panic disorder, and drug abuse or dependence. Of all the secondary psychiatric disorders analyzed, only antisocial personality disorder was more prevalent among male than among female alcoholics (these diagnoses are listed in the *Diagnostic and Statistical Manual of Mental Disorders, Third Edition* [American Psychiatric Association 1980]).

### Multiple Drug Abuse

Women who abuse alcohol also are likely to use and abuse other drugs, either sequentially or simultaneously (e.g., Beckman 1994). According to a 1991 survey of treatment facilities (Substance Abuse and Mental Health Services Administration 1993), 40 percent of women with alcohol problems were being treated in programs for alcohol and other drug abuse. Multiple drug abuse is a problem especially among younger women (under 35 years of age), who have a higher prevalence of marijuana, cocaine, and other drug abuse than do older women (Harrison 1989).

Many women with alcohol problems, including multiple substance abuse problems, receive treatment in chemical dependency rather than in alcohol-specific programs. However, women who are multiple substance abusers may require different treatment approaches and support services, depending on the kind and combination of drugs abused.

### Coping Mechanisms

Alcoholic women often have poorer coping mechanisms for dealing with stressful events than do nonalcoholic women (Gomberg 1993). Avoidant coping styles that involve denial or minimization of problems seem to be characteristic for women problem drinkers. Self-medicative use of alcohol may be a primary coping mechanism for these women. To improve treatment outcome and reduce the risk of relapse, coping skills training should be a part of women-sensitive treatment.

## Do Treatment Outcomes Differ for Women and Men?

Despite the perception by many health care providers that alcohol-abusing women are more difficult to treat than men, most reviews of the literature suggest that there are few differences in treatment outcome for women and men (e.g., Vannicelli 1984). Jarvis (1992) concluded that women have slightly improved treatment outcomes compared with men during the first 12 months after treatment, whereas men show greater improvement than women in long-term followup. This comparison is marred, however, by the heterogeneity of the treatment programs compared and the differences in length of followup between programs.

A recent analysis of more than 12,000 alcoholic patients in New Jersey, on the other hand, found that women were more likely to drop out of outpatient alcoholism treatment than men were (Mammo and Weinbaum 1993). Patients who did not complete treatment were less likely to have successful treatment outcomes (e.g., high rates of abstention or reduction of drinking) than were those who did complete treatment.

### Gender-Specific Success Rates of Different Treatment Approaches

Jarvis (1992) suggested that men and women may respond differently to different treatment types. For example, participation in group therapy may be associated with better treatment outcomes for men than for women (e.g., Cronkite and Moos 1984). Several explanations have been offered for this finding (Jarvis 1992):

- Women may prefer more individualistic settings to avoid the social stigma associated with drinking problems.
- Mixed-gender groups usually are composed primarily of men and therefore may ignore women’s issues.
- The group dynamics and styles of interaction of mixed-gender groups may be more agreeable to men.
- Group therapy sessions may preferentially satisfy certain social and affiliative needs for men that previously were satisfied by the drinking group, whereas women may desire social connections with individuals.

Sanchez-Craig and colleagues (1989) reported that when the treatment goal is moderation of drinking, women who are not severely dependent on alcohol are more successful than men. Women appear to achieve better results than men through a self-monitoring program that emphasizes behavioral self-control, and women and men were equally successful when working with a therapist to achieve moderation of drinking (Sanchez-Craig et al. 1991). Again, this may occur because social stigma is minimized in the self-monitoring treatment.

However, some studies do suggest that women do well in self-help groups, such as Alcoholics Anonymous (Gomberg 1993), perhaps because of women’s greater involvement in and connection to the social environment the self-help group provides.

## Women-Only Programs

In the past 10 to 15 years, numerous women-only programs have been implemented, based on the contention that women have different patterns of alcohol-related problems and different needs in treatment from those of men (Duckert 1987). The usefulness of these programs has been suggested, especially for women who have suffered (sexual) abuse or various traumas by men (Jarvis 1992).

There is as yet little research on the effectiveness of women-only programs compared with mixed-gender programs. In the only controlled study to date, Dahlgren and Willander (1989) compared women in a specialized female treatment program with patients in a traditional program. Women in both programs had received no previous treatment for alcohol problems and were in the early phases of alcohol dependence. The 2-year followup study showed better outcomes regarding alcohol consumption and social adjustment for patients in the women-only program.

One advantage of women-only treatment programs may be their ability to attract women to treatment earlier (Dahlgren and Willander 1989) or to attract women hesitant to attend traditional treatment programs. The notion that women-only programs recruit a different group of clients than mixed-gender programs is supported by Copeland and Hall’s finding (1992) that clients in a specialized women’s program were more likely to have dependent children, be lesbian, or have suffered sexual abuse in childhood than women in other programs.

On the other hand, the presence of women-only programs may cause mixed-gender programs in the same geographical area to pay even less attention to women’s treatment needs. This could reduce the overall quality of alcoholism treatment for women in mixed-gender programs (Reed 1987).

## Conclusion

Alcohol abuse in women may have risk factors and consequences that differ from those for men. If alcoholism treatment services are to be developed that truly meet the needs of women in general and of diverse subgroups, these factors as well as the differences in the gender roles and psychological identity development of women and men must be acknowledged and understood.

Recent theories emphasize as assets rather than as disadvantages for women’s psychological development the traditional qualities attributed to women (e.g., cooperative, passive, supportive, giving, emotional, and vulnerable) and their need for social connections (Miller 1984). Therapists sensitive to women’s needs have begun to apply these ideas to treatment for female substance abusers, focusing especially on the importance of building satisfying and supportive social relationships in women’s lives.

But not only the development of new women-sensitive treatment programs is necessary to improve alcoholism treatment for women. It also is imperative that the effectiveness of these new programs be evaluated in rigorously designed scientific studies, thus closing the gap between beliefs and facts about women-sensitive treatment.

## Footnotes

1 Terms such as “women with alcohol problems” and “female problem drinkers” used throughout this article refer to women whose drinking patterns have caused negative social, personal, or medical consequences.

2 Because the exact level of alcohol consumption required to cause adverse medical or social effects cannot be determined, the term “excessive” may represent different drinking levels for each individual.
